# Genetic Appraisal of RAAS-Associated SNPs: REN (*rs*16853055), AGT (*rs*3789678) and ACE (*rs*4305) in Preeclamptic Women Living with HIV Infection

**DOI:** 10.1007/s11906-023-01292-y

**Published:** 2024-02-27

**Authors:** Annelene Govindsamy, Shoohana Singh, Thajasvarie Naicker

**Affiliations:** Optics and Imaging Centre, Doris Duke Medical Research Institute, College of Health Sciences, University of KwaZulu-Natal, Durban, South Africa

**Keywords:** HIV, Preeclampsia, Hypertension, *rs*4305, *rs*16853055, *rs*3789678

## Abstract

**Purpose of Review:**

The primary goal of this review article was to determine whether the three RAAS-associated SNPs, Renin-rs16853055, AGT-rs3789678 and ACE-rs4305 are genetically linked to the development of hypertension in preeclampsia. The secondary goal was to establish if there was a link between these SNPs and HIV infection.

**Recent Findings:**

There is a paucity of findings related to the aforementioned SNPs and preeclampsia. There are no recent findings on the rs16853055 renin polymorphism. The rs3789678 angiotensinogen polymorphism correlated significantly with gestational hypertension. The rs4305 ACE polymorphism showed no significant association with the development of pregnancy-induced hypertension.

**Summary:**

There are conflicting findings when determining the relationship between ethnicity and the predisposition of preeclampsia and hypertension in relation to the discussed RAAS-associated SNPs. To date, the association between RAAS-associated SNPs and preeclamptic women co-morbid with HIV in South Africa has revealed that certain alleles of the AGT gene are more prominent in HIV-infected PE compared to normotensive pregnant HIV-infected women.

## Introduction

A healthy pregnancy outcome may be attributed to the homeostasis of the renin-angiotensin-aldosterone system (RAAS) [1] and its signalling cascade between mother and foetus [2]. During normal pregnancy, angiotensin-converting enzyme (ACE) is the only component of the RAAS that is downregulated [3]. However, in pathological complications such as preeclampsia (PE), the RAAS is dysregulated [1] since the levels of renin, angiotensin I (Ang I) and angiotensin II (Ang II) are reduced [4]. Preeclampsia is primarily defined by new onset hypertension (systolic blood pressure ≥ 140 mmHg or diastolic pressure ≥ 90 mmHg) following 20 weeks of gestation, with or without proteinuria (≥ 30 mg/mmol obtained from a 24-h urine test) [5]. Utero-placental dysfunction and maternal organ malfunction (kidney, liver and haemostatic system) may/may not be present [6, 7]. Multiple factors influence the prevalence of PE such as parity, ethnicity, economic and social constraints and geographic location [5, 8]. In 2020, 95% of maternal mortality predominated in low-to-middle income countries [9].

Furthermore, maternal deaths emanating from PE development are high with an incidence of 2–10% globally [10]. Approximately 1.8–16.7% of these deaths have been recorded in developing nations as opposed to 0.4% in developed populations [11]. There were 287,000 deaths recorded during and after delivery [9], with 86% of these deaths emerging from Southern Asia and sub-Saharan Africa [12]. Human immunodeficiency virus infection/acquired immunodeficiency syndrome (HIV/AIDS) is a primary public health concern, particularly in Africa [13]. Although HIV/AIDS is the primary cause of maternal mortality in South Africa (SA), epidemiological studies have revealed that PE is the direct cause of maternal mortality accounting for 14.8% of all deaths [14]. In SA, the province of KwaZulu-Natal has a maternal death rate of 13.35% related to hypertensive disorders of pregnancy (HDP) [15]. Additionally, the prevalence rate of HIV infection and PE is high in SA and thus is a serious public health challenge [16, 17]. Against this backdrop, the synergy of HIV infection and PE warrants urgent attention; hence, this narrative review appraises the genetic polymorphisms of RAAS in pregnancy.

**Fig. 1:**
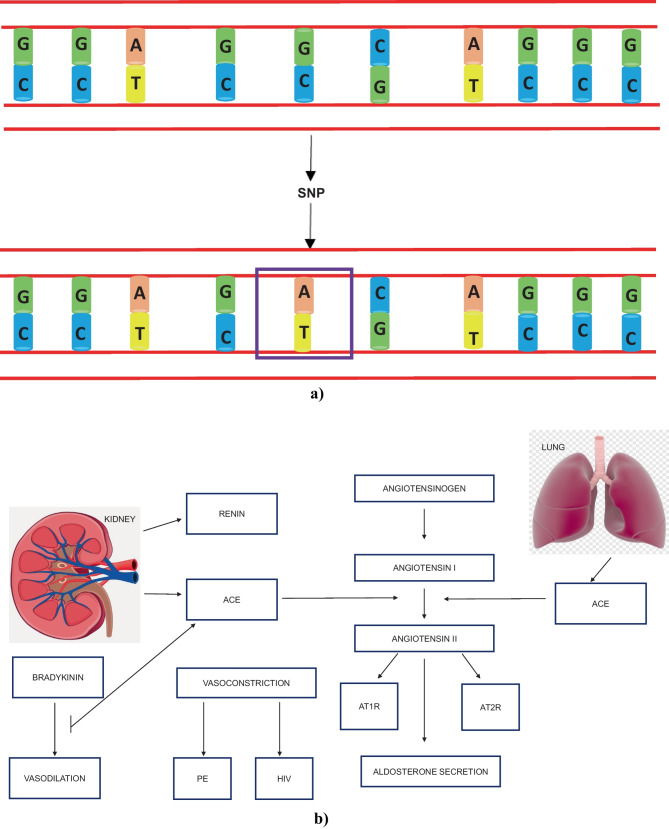
*Single nucleotide polymorphisms and the RAAS.* **a** Double-stranded DNA showing the outcome of a genetic mutation (SNP) (adapted from [26]). **b** The RAAS pathway is displayed in conjunction with prominent functions/effects of certain analytes (adapted from [27]). (ACE, angiotensin-converting enzyme; AT1R, angiotensin II type 1 receptor; AT2R, angiotensin II type 2 receptor)

Single nucleotide polymorphisms (SNPs) are frequently occurring DNA sequence variations that culminate in genetic aberrations of a single nucleotide within the genomic sequence [18] (Fig. 1a). Single nucleotide polymorphisms may represent a genetic avenue to determine a women’s genetic predisposition to an illness or a condition as well as their response to therapeutics [19•]. Both the identification and subsequent characterisation of a multitude of SNPs are required prior to their frequent use as a genetic gold standard to identify PE predisposition. The frequencies of SNP alleles vary drastically amongst different ethnicities within the human population, suggesting that many investigations can be performed on different populations to attain a similar goal. There is a lack of research conducted on polymorphisms that predispose hypertension amongst individuals of African ancestry. Therefore, extensive genetic studies are required to assess the relationship between African populations and polymorphisms that increase susceptibility to hypertension and/or other disease development [20••].

This review firstly outlines the RAAS and its associated components in normal and then in PE co-morbid with HIV infection. Secondly, this manuscript highlights RAAS-associated SNPs and their involvement in the development of hypertensive associated co-morbidities. The RAAS-associated SNPs are potential candidates for gene-related studies focused on both pregnancy-induced hypertension (PIH) and hypertension per se [21••].

## The RAAS and its Components

The RAAS regulates blood pressure and water-electrolyte equilibrium via endocrine and intravascular pathways [22•]. Of note, dysregulation of components of RAAS has been implicated in both the first and second stage of PE development [23••, 24•]. The RAAS is composed of a collection of hormones and enzymes that associate with each other (Fig. 1b), namely, renin (REN), angiotensinogen (AGT), angiotensin-converting enzyme (ACE), angiotensin I (Ang I), angiotensin II (Ang II), angiotensin receptors, aldosterone, aldosterone receptors and the *mitochondrial assembly protein 1* receptor ( MAS) receptors [25].

*Renin* (*REN*) is produced, stored and secreted by the juxtaglomerular cells of the kidney [28]. It is an essential mono-specific enzyme that consists of 406 amino acids in addition to a pre-segment and pro-segment of 20–23 and 43–47 amino acids, respectively [29]. It is composed of 12.5 kb of DNA consisting of ten exons [30] and eight introns [31].

Its precursor form, pro-renin, is produced in the adrenal gland, testis, placenta and eye and is activated by enzymes via receptor binding action [32]. Renin may also serve as a hormone due to its signalling function. It manifests its action by decreasing arterial blood pressure, salt chloride levels and sympathetic nervous system activity [33]. Subsequently, REN hydrolyzes AGT (produced in the liver) [34], to angiotensin I (Ang I) via its leucine-valine bond [35]. Renin cleaves the N-terminal of AGT which results in the formation of angiotensin I [36].

*Angiotensin-converting enzyme (ACE)—*ACE consists of 25 introns and 26 exons [37]. The human form of this gene is located on chromosome 17q23.3 [37]. It is produced in the endothelial cells of the lungs and epithelial cells of kidneys where it converts inactive Ang I to active angiotensin II (Ang II). ACE cleaves two amino acids from the C-terminal of angiotensin I to make the peptide angiotensin II. Also, ACE has a degradative effect on active bradykinin (BK), which plays an important role in controlling blood pressure (Fig. 1b) [27, 38, 39].

*Angiotensin I—*Notably, angiotensin I is synthesised via REN by cleaving ten amino acids from its N-terminal [40]. Further, it is also a source of several activated angiotensin peptides.

*Angiotensin II*—Notably, Ang II is a powerful vasoconstrictor responsible for the elevation of blood pressure, thereby increasing the pulse speed of the cardiovascular system and triggering plasminogen activator inhibitor proteins thus elevating pro-thrombotic capacity [41]. Although the half-life of Ang II is 30 s [42], it may be converted to angiotensin III (Ang III) by the action of aminopeptidase A on erythrocytes [43]. Angiotensin-converting enzyme-2 (ACE2) plays an essential role in the RAAS as it has opposing functions to Ang II [44]. Therefore, both ACE and ACE2 possess significant functions; in that, ACE indirectly elevates blood pressure in a volume-depleted milieu [45] whilst ACE2 is beneficial for the kidney [46]. Moreover, Ang II activates the adrenal gland cortex to release aldosterone [47].

*Aldosterone*—Aldosterone exerts its function by maintaining sodium-potassium homeostasis. This is achieved by activating the proximal convoluted tubules of the kidney, culminating in elevated sodium reabsorption, thus preserving sodium concentration with concomitant release of potassium [48]. Also, it stimulates the hypothalamus by triggering the thirst reflex, with consequent release of anti-diuretic hormone (ADH) to limit urinary loss [49].

*Angiotensin receptors*—There are four angiotensin receptors:

(i) *Angiotensin II-type 1 receptor* (AT1R) occurs in the renal vasculature, glomerular mesangium, interstitial cells and proximal tubules. This receptor functions via signalling pathways to increase intracellular calcium whilst also causing vasoconstriction, sympathetic activity and aldosterone release [3] (Fig. 1b).
(ii) *Angiotensin II-type 2 receptor* (AT2R) shares a similar location as AT1R. It inhibits cell growth and initiates apoptosis, causing vasodilation thus promoting foetal development [3]. It is important to study both receptors collectively as they are both derivatives of the seven-transmembrane G-protein-coupled receptor bearing equal affinity to Ang II [50].
(iii) *Angiotensin II-type 3 receptor* (AT3R)—there is a dire limitation of available data warranting further research.
(iv) *Angiotensin II-type 4 receptor* (AT4R) bears an increased affinity to membrane binding loci for [^125^I] Ang IV peptide. Further, these receptors are found mainly in the brain and to a small extent, in the vasculature, kidneys, heart and adrenal glands. Additionally, they function in the regulation of blood flow, vasodilation and improved cognition [51–53].
(v) The *mitochondrial assembly protein 1* receptor (MAS1 oncogene) is the primary receptor for RAAS-secreted Ang 1-7 as it is structurally similar to several other G-protein-coupled receptors [52]. When stimulated by binding to Ang (1-7), it opposes the effects of Ang II-stimulated-angiotensin-receptors. The MAS receptor is expressed on the endothelium and binds to Ang (1-7), resulting in localised redox balance, reduced oxidative stress in addition to anti-fibrosis. Its location includes the vasculature, brain, kidneys and the heart [54].

## RAAS and HIV Infection

**Fig. 2:**
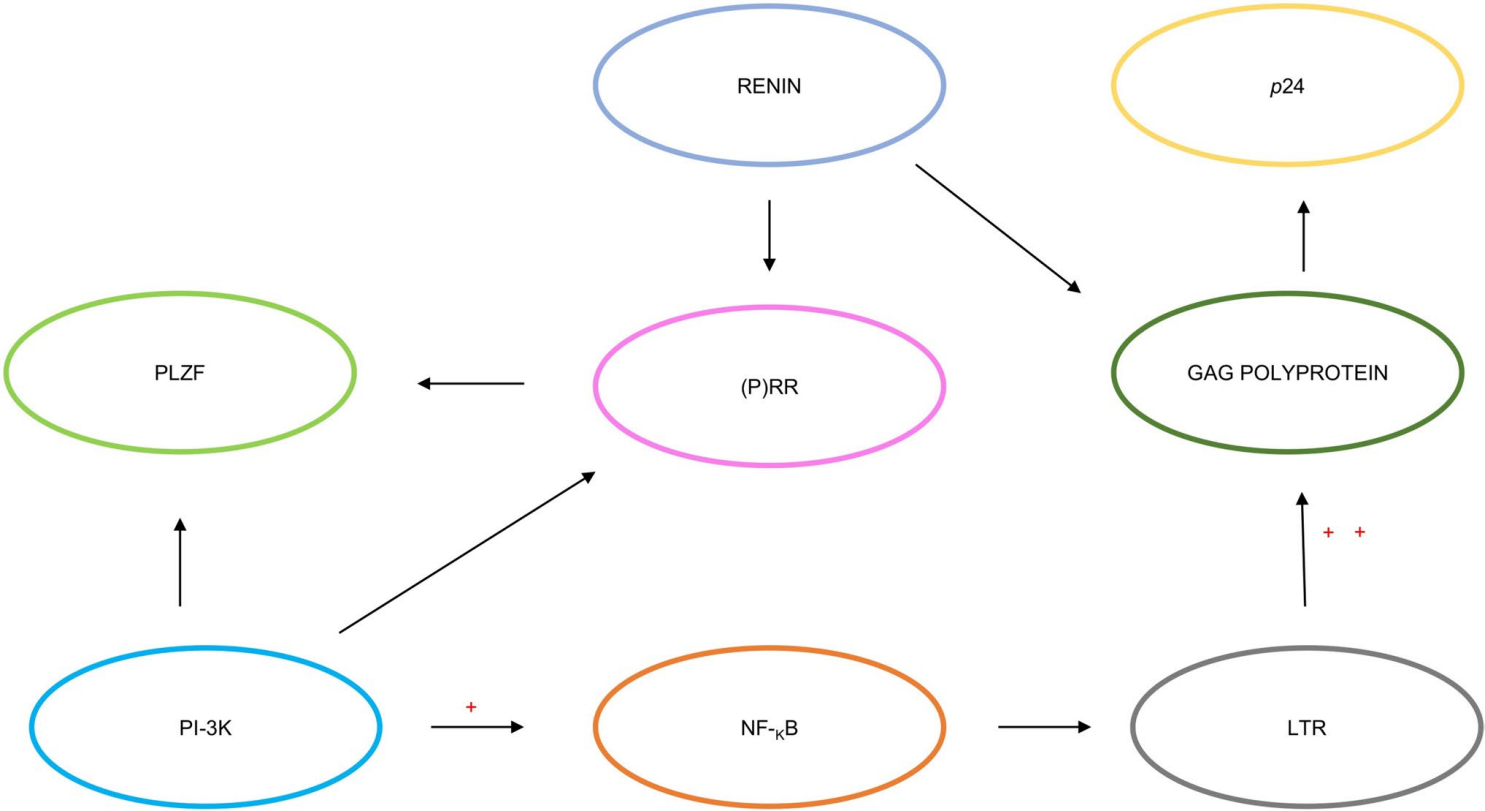
*The role of renin in HIV infection* (adapted from [56••]). The interaction between HIV infection and REN is established via receptors and pathways, resulting in the synthesis of *p24*, a core HIV protein. ((P)RR, pro-renin receptor; PLZF, promyelocytic leukaemia zinc finger; PI-3 K, phosphoinositide 3-kinase; NF-_K_B, nuclear factor kappa-light-chain-enhancer of activated B cells; LTR, long terminal repeat). +Increases function, ++accelerates synthesis of polyproteins

Hypertension and inflammation are both triggered by the direct effect of HIV infection on the RAAS, linked to T cell activation [55••]. Of note, REN stimulates HIV replication within T cells by triggering the activation of both nuclear factor kappa-light-chain-enhancer of activated B cells (NF-_K_B) and phosphoinositide 3-kinase (PI-3 K) pathways [56••]. Therefore, the emergence of metabolic syndrome and hypertension in people living with HIV (PLWH) is influenced by ongoing immunological activation [55••]. During HIV infection, serum ACE levels are increased compared to control individuals [57]. The PI-3 K and NF-_K_B pathways are activated in addition to the pro-renin receptor [(P)RR] [56••]. This activation, in conjunction with the cleaving of HIV Gag-polyproteins, culminates in an increased rate of HIV replication in T cells via the REN protein. The association of REN with (P)RR culminates in its attachment to promyelocytic leukaemia zinc finger (PLZF) and its consequent nuclear translocation [56••]. Subsequently, the PLZF-stimulated pathways increase NF-_K_B function, culminating in its binding to the LTR promoter region, thus accelerating the synthesis of the Gag-polyprotein. Gag polyproteins are then cleaved by HIV protease (Hpr) and REN, leading to the production of pro-viral proteins, particularly, HIV’s *p*24 (Fig. 2).

Despite HIV infection neutralising immune exaggeration of PE [58], some studies have reported HDP predisposition [59]. It is widely accepted that highly active antiretroviral therapy (HAART) increases PE risk by immune restoration, thus influencing the RAAS downstream [60, 61]. The function of HAART during HIV infection is to hinder viral replication, culminating in the suppression of viral transmission between the mother and child [62]. Additionally, HAART induces the pro-inflammatory profile of the HIV-infected mother, thus triggering the development of HDP [63–65••

## RAAS in the Synergy of HIV Infection and Preeclampsia

Trans-activator of transcription (*Tat*), a regulatory protein of HIV-1, increases viral infectivity [66] and is rich in both lysine and arginine thus resembling the vascular endothelial growth factor (VEGF) sequence [67]. Consequently, VEGF’s function is mimicked by *Tat*, upregulating angiogenesis, and the expression of αvβ3 and α5β1 integrin and endothelial cell (EC) adhesion [67, 68]. These can further bind to angiogenic factors that play a role in decidualization [69]. It was shown that both endothelial nitric oxide synthase (eNos) expression and endothelium-dependent vasorelaxation were reduced by the *Tat* protein. Further, these researchers revealed that *Tat* was involved in the development of coronary artery disease, which is an outcome of PE in later life [70]. Additionally, *Tat* induces the expression of both vascular cell adhesion protein-1 (VCAM-1) and intercellular adhesion molecule-1 (ICAM-1), inferring a potential mechanism via which HIV-1 intensifies endothelial injury as well as atherosclerosis [71, 72].

## Genetic Appraisal of RAAS

Genetic aberrations of individual components of the RAAS result in aberrant physiological manifestations and subsequent hypertension [73]. However, hypertension is a condition that is multifaceted emanating from both genetic as well as environmental factors [74]. Single nucleotide polymorphisms (SNPs) are associated with the pathophysiology of several diseases, including HIV/AIDS [75–77]. Of note, the C-C chemokine receptor type 5 delta 32 promoter SNP (CCR5Δ32) is prominent in people of African American and European ancestries [78••]. Interestingly, this SNP could serve as a biomarker for the early diagnosis of HIV infection [78••]. Also, pharmacogenetic studies of these SNPs in HIV infection may provide therapeutic intervention [79]. In relation to PE, studies have revealed the association between SNPs and the risk of PE development [80]. However, there is a demand for more geneticists to holistically understand the genetic role of SNPs in the onset of PE [81]. Ultimately, SNPs could serve as genetic contributors to disease onset thereby aiding researchers in determining the etiology of certain diseases and infections [82]. Taken together with non-genetic contributors, genetic variation within the RAAS may predict one’s risk of developing hypertension [83••]. In the succeeding paragraphs, we highlight the pertinent findings of renin, AGT and ACE SNPs in relation to PE, PIH and hypertension.

### Renin (REN) Panel

REN is an eminent candidate gene for the development of PE [84••]. However, a limited number of studies have determined the association between this gene and PE [84••]. Furthermore, no positive associations were noted between maternal risk of PE development in relation to foetal REN haplotypes [84••]. Additionally, these researchers could not distinguish between paternally or maternally inherited REN when establishing the association of foetal REN with maternal AGT within study groups. In a study conducted in Chile, no association between PE and variants of the REN gene in offspring was reported [85]. In contrast, a strong correlation between REN (*rs*11240688) and AGT (*rs*11122576 G>A) foetal polymorphism and PE development was noted in a North Indian population study [86]. This observation indicates a strong association between foetal genotypes of REN and AGT that give rise to the stimulation of maternal RAAS and the disruption of angiogenesis, thereby triggering maternal PE.

In recessive models in central China, both foetal and maternal REN (*rs*5707) correlated strongly with PE and eclampsia development [87]. Contrary to these findings, SNPs of the REN gene (*rs*5705, *rs*1464818 and *rs*3795575) revealed no association with the development of PE [84••]. A Spanish population study of non-pregnant women who carried the GG phenotype of the REN (*rs*5707) polymorphism revealed a strong correlation with hypertension development. However, the mechanism by which PE is governed emanating from the REN (*rs*5707) polymorphism is yet to be discovered [87]. In both Japanese [88] and Northern Chinese women [89], it was shown that there was no association between the development of hypertension and the REN (*rs*5707) polymorphism. Despite an absence of relationship between the REN (*rs*5707) and REN (*rs*2368564) polymorphisms in a central China population study, Mexican women showed SNPs of the REN gene with strong association to an increased risk of hypertension development [90].

An association between maternal AGT and foetal-REN was documented in animal models [84••]. The mating between transgenic mice who expressed human AGT and human REN culminated in pregnant females who displayed a temporary increase in blood pressure in late phases of pregnancy emanating from the release of human REN from the placenta into the maternal circulation [91•]. This infers that the release of human REN in the placenta by paternal genes could enter and associate with maternal AGT in the circulation, thus, activating PE symptoms [91•]. These results were synonymous with preeclamptic transgenic rodent models [92]. Therefore, these findings collectively suggest that ethnicity plays a role in the pathogenesis of PE and hypertension emanating from genetic aberrations that are unique to a specific population. There is a paucity of data on REN (*rs16853055*) polymorphism. Purkait et al. showed that this polymorphism had no association with diabetic nephropathy in participants of Indian ancestry [93]. Since this SNP has not yet been analysed within the realm of HDP co-morbid with HIV in sub-Saharan Africa, particularly South Africa, extensive research is required to enhance our understanding of its role as a genetic contributor to disease development as well as its associated functional properties.

### Angiotensinogen (AGT) Panel

The production of AGT is promoted by oestrogen, culminating in increased levels in circulation during the first 80 days of gestation [23••]. Angiotensinogen gene polymorphisms may increase plasma levels in PE [94]. Furthermore, the T allele of AGT may probably be a major contributor to the onset of PE [95••]. Despite the fact that AGT levels are comparable between normal pregnancy and PE, the high-molecular-mass form of this gene remains relatively higher during PE [23••]. Interestingly, in this form, its prevalence is less than 5% in non-pregnant women [96]. However, under normal pregnancy conditions, it increases to 16%, attributed to utero-placental release [23••]. Moreover, a higher AGT level (28%) was observed in women who carry the gene for PIH [23••]. Two genes were associated with the development of hypertension in the Han Chinese population, namely, AGT (*rs3789678*) and ACE (*rs4305*) [83••]. However, the AGT (*rs3789678*) polymorphism in both Caucasian and African-American populations did not yield the Hardy Weinberg equilibrium [97], thus inferring population-specific discrepancies.

In comparison to normotensive pregnant women, PE women display a higher concentration of AGT in its oxidised form [98]; thus, it could infer antioxidants that lead to PE development. In pregnant murine models, the overexpression of AGT led to an unmaintained plasma volume overload [99]. This infers that these mice do not have the genetic capacity to upregulate the expression of REN in the nephron [99]. Whether this finding extrapolates to human PE is unknown [23••]. Aung et al. reported that the TT genotype and the T allele of the AGT gene (*M235T*) were higher in the synergy of HIV-infected PE than normotensive HIV-infected women. Additionally, in the latter group, there was a higher distribution of AGT particularly, the MT genotype in comparison to those who were preeclamptic and infected with HIV (19% vs. 10%; *p* = 0.03) [95••]. Further, there was no association between the AGT (*M235T*) and REN (*C-5321 T*) polymorphisms in the normotensive groups when investigating early-onset PE (EOPE). Furthermore, the MM genotype of AGT was only present in the normotensive group [95••]. These authors proposed that the T allele and TT genotype of the *M235T* polymorphism predisposed South African women of African ancestry to developing PE twofold higher than normotensive pregnant women who displayed the MT, MM and M alleles, independent of HIV status. An association between PE and the AGT gene (*M235T*) in women of Greek descent in the North-Western region of Greece [100], Iran [101], and Chinese women [102] was noted. Furthermore, a correlation between the AGT-*M235T* gene polymorphism and chronic hypertension was also recorded in Caucasian-Dutch women [103].

In contrast, AGT-*M235T* polymorphism occurs in South African women of African ancestry [104••], whilst Caucasian and African-American women had no association with the development of PE [105]. Similarly, in North India it was established that the AGT-*T704C* polymorphism did not contribute to the development of PE [106]. These variations may be attributed to different ethnicities, different sources of DNA, and both reagents and instruments employed in the study and the sample size [95••]. The Genetics of PE Collaboration (GOPEC) study revealed inconclusive findings when comparing both foetal and maternal AGT genotypes in relation to the development of PE [107]. In relation to HIV infection, a relationship was established between the T allele of the AGT polymorphism in preeclamptic HIV-infected women and in normotensive subjects [95••]. This, however, was absent in HIV-uninfected participants, indicating that HIV status did not contribute to PE development, contrary to what was reported for the AGT-*M235T* polymorphism.

In Romanian women, both the *M235T* and AGT-*174 M* polymorphisms were associated with the predisposition to early-onset PE (EOPE) rather than late-onset PE (LOPE) [108]. Urinary AGT represents a biomarker for the upregulation of RAAS and is subsequently increased during PE and gestational hypertension (GH) [109]. Therefore, the activation of the RAAS can be dysregulated intra-renally emanating from endotheliosis and hypertension during pregnancy [109]. Consequently, this enhances the pathogenesis of both hypertension and renal injury.

Researchers at the University of Norway demonstrated a strong correlation between PE and maternal AGT [84••]. Furthermore, these results were correlated with AGT A-Met-Thr (G1035A-Thr174Met-Met235Thr) in preeclamptic French-Canadian Caucasians [110] who had an increased risk of disease development compared to normotensive subjects [110]. Contrary to this finding, the GOPEC study revealed no association between PE and haplotypes of maternal AGT when investigating the relationship between 536 foetal triads (mother-father-child) and 398 maternal triads (grandmother-grandfather-mother) of British descent [107]. The variations of AGT (*rs7079*) revealed staggering differences when evaluating PE prevalence, particularly the severe form and is, therefore comparable to the mild form of PE [86]. South Africans of African ancestry have salt-sensitive hypertension [111] and thus could be less accommodating to RAAS inhibitors [20••]. Therefore, the presence or absence of RAAS-associated polymorphisms could influence the outcome of anti-hypertensive therapy [20••]. This was shown by Woodiwiss and co-workers where AGT SNP genotypes had varying responses to ACE inhibitors in individuals of African ancestry [112]. From these findings, one may elucidate that ethnicity may/may not genetically predispose hypertension development.

### Angiotensin-Converting Enzyme (ACE) Panel

Angiotensin-converting enzyme, an indirect regulator of blood pressure, may involve insertion/deletion (ACE I/D) polymorphisms in PE [113–115]. Preterm birth is associated with an insertion/deletion of ACE polymorphisms [116]. However, these results may be based on ethnicity [116] as pregnant women in Brazil showed a correlation between ACE polymorphisms and PE [113]. In contrast, other groups have shown no association between these polymorphisms and PE development in the general Brazilian population [117, 118].

Studies performed in other ethnic groups, namely, South African, Chinese, and Caucasian populations also show no positive correlation with the development of PE [104••, 119, 120]. However, there was a strong association between elevated PE risk and the D allele in Turkish, South-East Iranian, Mexican and Egyptian women [115, 121–123]. A strong association was noted between EOPE and the DD genotype in Egyptian women [123]. In pregnant Chinese women who displayed the D allele, both renal dysfunction and severe proteinuria were a common anomaly [120]. Additionally, preeclamptic Italian women who displayed the DD genotype had increased pulsatility index values in the umbilical artery at the 16th, 20th and 24th week of gestation in comparison with those who displayed the II and ID genotypes [124].

ACE polymorphisms may negatively impact both serum and tissue enzyme levels, resulting in PE development [125]. One such polymorphism is the ACE *(rs4343*) which is significantly associated with PE in Iranian women [125]. However, there was no evidence of a positive association between PE development and the ACE I/D alleles in the same population [125]. There are currently only two investigations that have studied the influence of the ACE (*rs4343*) polymorphism in the development of PE [126, 127]. Evidently, in Han Chinese women, foetal ACE (*rs4343*) was associated with the development of PE. However, this differed in maternal ACE (*rs4343*) [126]. A European-based study revealed a directly proportional relationship between ACE (*rs*4305) and hypertension [128]. Elevated ACE activity resulted in dysregulated angiogenesis and placental circulation, consequently leading to adverse gestational outcomes [129]. Contrary to this finding, the inhibition of ACE culminated in endothelial apoptosis [130]. Interestingly, Gathiram and Moodley showed that ACE polymorphisms did not contribute to the development of PE [131••] (Table 1).

**Table 1:** Pertinent findings on three RAAS-associated panels, namely, REN, AGT and ACE

Gene/SNP	Title	Main findings	Demographics	Reference
RENIN (*rs*11240688)	Polymorphisms in renin-angiotensin-aldosterone system and vascular endothelial growth factor may cross talk in preeclampsia: a pilot study of maternal and fetal dyads in Indian population.	Strong association between REN (*rs*11240688) and the AGT (*rs*11122576 G>A) foetal polymorphism and PE	North Indian ancestry	[86]
RENIN (*rs*5707)	The C4280A (*rs*5705) gene polymorphism of the renin (REN) gene is associated with risk of developing coronary artery disease, but not with restenosis after coronary stenting.	Predisposes women to an increased risk of developing hypertension	Mexican ancestry	[90]
AGT (*rs*3789678)	Renin–angiotensin–aldosterone system gene polymorphisms in gestational hypertension and preeclampsia: a case–control gene-association study	The AGT *rs*3789678 polymorphism had a strong correlation with GH but not with PE. The TT vs. TC genotype could possibly increase the risk of developing GH	Chinese ancestry	[21 ••]
AGT (*M235T*) AGT (MT)	Association of gene polymorphisms of four components of renin-angiotensin-aldosterone system and preeclampsia in South African black women.	The T allele of the AGT (*M235T*) polymorphism was higher in HIV-infected women with PE. A greater distribution of AGT (MT) genotype compared to preeclamptic HIV-infected women	African ancestry in South Africa	[95••]
AGT (*rs*7079)	Polymorphisms in renin-angiotensin-aldosterone system and vascular endothelial growth factor may crosstalk in preeclampsia: a pilot study of maternal and foetal dyads in Indian population.	The AGT (rs7079) polymorphism revealed a significant difference when assessing the prevalence of severe PE	North India (Indo European origin)	[86]
ACE (*rs*4305)	Association of hypertension drug target genes with blood pressure and hypertension in 86,588 individuals.	Directly proportional relationship between the ACE *rs*4305 polymorphism and hypertension	European ancestry	[128]
ACE (*rs*4343)	The gene variants of maternal/fetal renin-angiotensin system in preeclampsia: a hybrid case-parent/mother-control study.	Foetal ACE (*rs*4343) was associated with the development of PE	Han Chinese ancestry	[126]

## Conclusion

In summary, this review article appraises RAAS-associated polymorphisms in relation to PE co-morbid with HIV infection in this ART era. We report contradictory findings of REN (*rs5707*) polymorphism and the risk of PE development. The AGT (*M235T*) polymorphism has however been widely associated with hypertensive development across varying ethnicity. Nonetheless, AGT (M235T) variants have also been reported to have no association with PE development. Lastly, the ACE (*rs4343*) polymorphism is associated with the development of PE independent of ethnicity. Given that many of these SNPs have not been investigated in relation to preeclampsia (PE) and HIV infection, extensive research is required to focus on the genetics of PE. In sub-Saharan Africa, researchers should focus on studies that include PE co-morbid with HIV in women of African ancestry in South Africa as the burden of HIV and PE prevalence is high.

## Future Recommendations

SNPs of all components of the RAAS are warranted particularly in low-middle-income countries.

## Data Availability

All relevant data is available within the text.
